# A sub-chronic feeding study of dual toxin insect-resistant transgenic maize (CEMB-413) on Wistar rats

**DOI:** 10.1371/journal.pone.0285090

**Published:** 2023-08-09

**Authors:** Ayesha Liaqat, Ibrahim Bala Salisu, Allah Bakhsh, Qasim Ali, Ayesha Imran, Muhammad Azam Ali, Abdul Munim Farooq, Abdul Qayyum Rao, Ahmad Ali Shahid

**Affiliations:** 1 Centre of Excellence in Molecular Biology, University of the Punjab, Lahore, Pakistan; 2 Department of Animal Science, Faculty of Agriculture, Federal University Dutse, Dutse, Jigawa State, Nigeria; 3 Department of Molecular Biology, Virtual University of Pakistan, Lahore, Pakistan; University of Tennessee, UNITED STATES

## Abstract

Genetically modified (GM) crops expressing insecticidal crystal proteins are widely accepted worldwide, but their commercial utilization demands comprehensive risk assessment studies. A 90-day risk assessment study was conducted on Wistar rats fed with GM maize (CEMB-413) expressing binary insect-resistant genes (c*ry1Ac* and c*ry2Ab*) at low (30%) and high (50%) dose along with a control diet group. The study used fifty Wistar rats randomly distributed in five treatment groups. Our study revealed that compared to controls, GM diet had no adverse effects on animal’s health, including body weight, food consumption, clinical pathological parameters, serum hormone levels and histological parameters of testes and ovaries of rats. Differences were observed in transcripts levels of fertility related genes, but these were independent of treatment with GM diet.

## 1. Introduction

Maize is a valuable crop for animal feed because of its availability, cost effectiveness and nutritional value. Since most of the yield losses in maize occur due to insect pest attack, The insecticidal *cry* genes from *Bacillus thuringiensis* encode Cry proteins that show toxicity against a variety of insects belonging to the Lepidoptera, Coleoptera and Hymenoptera orders and nematodes [1–3]. Therefore, transgenic crops producing Cry proteins have emerged as a possible solution to limit insect damage and have been commercially grown on large scale worldwide. Several crops including maize, rice, cotton, sugarcane, tomato, potato, eggplants, alfalfa and tobacco have been engineered with insecticidal *cry* genes and some of them have been commercialized [4]. Since first commercialization, the adoption of GM maize has been grown to currently represent approximately 31% of global acreage [5]. This increased adoption of GM crops has relevant benefits including, increased farm productivity and reduced need for application of chemical insecticides [6].

Despite the attractive benefits of GM crops, yet there are concerns about their safety [7, 8]. Concerns associated with GM food consumption include immunotoxicity development, change in gene expression, reproductive toxicity and other unintended effects [9–11]. Consequently, it should be the prime focus of competent authorities to guarantee the biosafety of new biotechnological events before their commercialization [12]. *Rattus norvegicus* (Wistar rat) is a widely used animal model to assess the potential effects of GM crops on human health, because they show 95% genetic homology with humans and can mimic human disease [13]. Ninety day-feeding trials are recommended by scientific regulatory and monitoring bodies to assess the possible toxicity of GM crops on human health [14, 15]. The majority of these studies revealed no adverse effect of GM crops consumption on animal health, including fertility-related parameters [16, 17], sex hormonal profile [18–20], haematological factors [21, 22], feed intake [21, 22], histopathology [20, 23, 24] and growth parameters [16, 20, 25]. However, opposite views also exist as some studies experimentally indicated adverse effects on fertility [26, 27], immunity [28], growth [29], hepatorenal parameters [30–32], haematological parameters [31, 33] and reproductive effects [34]. Additionally, very limited studies have been conducted to assess the effect of GM feed on the expression levels of fertility related genes. This is important considering that dietary supplementation irrespective of GM material participation can alter gene expression in animals and also have great impact on fertility and fitness of organisms even before the breeding process [35]. The current study was conducted to evaluate the possible effects of transgenic maize (CEMB-413) producing Cry1Ac and Cry2Ab insecticidal proteins on Wistar rat health, reproduction and fertility related parameters.

## 2. Material and methods

### 2.1 Bioethics and GM material

The established guidelines of the European Food Safety Authority (EFSA) and the Organization for Economic Cooperation and Development (OECD) were followed to carry out the current study on rodents. The animal facility at the Centre of Excellence in Molecular Biology (CEMB), University of the Punjab (Lahore, Pakistan), was used for this purpose. The study was approved by the Institutional Animal Ethics Committee (IAEC) of the institute. GM maize (CEMB-413) seed expressing Cry1Ac and Cry2Ab proteins were used as test material. The seed of the same non-GM maize (near-isogenic line) was used as reference/control. Transgenic and control seeds were obtained from the seed bank of the CEMB.

### 2.2 Composition of the diet

Diet for the standard group was formulated according to a procedure previously optimized at CEMB. Prior to diet formulation, the maize grains (both the transgenic and conventional isoline) were sterilized using 5% sodium hypochlorite and were further grounded to flour using a 50 number mechanical grinder (Pak Agro Engineers, Okara, Pakistan) for diet preparation. The ingredients of diet for both test and control groups were mixed thoroughly to ensure homogeneity. An equal number of grounded seeds of GM maize (CEMB-413) and its conventional isoline were added into other ingredients at the level of approximately 30% and 50% (w/w). Vitamins, salts and minerals were added in experimental diets in equal concentration to ensure suitable supply of nutrients. Different experimental diets (Table 1) were prepared containing standard diet, 30% non-GM and 30% GM maize diet, 50% non-GM and 50% GM maize diet.

**Table 1:** Composition of experimental diets.

Ingredients (%)	Dietary treatments				
	Control	30% non-GM	30% GM	50% non-GM	50% GM
Maize	26	**30**	**30**	**50**	**50**
Barley	26	25	25	15	15
Wheat	26	22	22	12	12
Fish meal	17.4	18	18	18	18
Sunflower Oil	4.3	3	3	3	3
Vitamins	0.3	1	1	1	1
Calcium Carbonate	-	0.5	0.5	0.5	0.5
Sodium Chloride	-	0.5	0.5	0.5	0.5

### 2.3 Molecular testing and compositional analysis of diet

The maize seeds CEMB-413 and its conventional counterpart were analysed to detect the presence of *cry1Ac* and *cry2Ab* by polymerase chain reaction (PCR) using gene specific primers. Cry1Ac and Cry2A proteins were also detected using an antibody specific Enzyme-linked immunosorbent assay (ELISA) using commercially available kits (Envirologix,catalog # AP003 for Cry1Ac and # AP005 for Cry2A. In addition, the presence of aflatoxins (AFG1, AFG2, AFB1, AFB2) in transgenic and control maize grains were assessed via an ELISA kit (Bio Shield Total, Cat No. B1648). The proximate compositions of diets was evaluated as described earlier by the AOAC [36].

### 2.4 Animals and housing

A total of 50 Wistar rats (25 males and 25 females, aged 5–6 weeks old at the start of the experiment) were kept at the CEMB animal house. The animal’s distribution and their housing were as described by Salisu et al. [37, 38] in earlier studies. The procedures of housing and treatments were approved by the ethics committee of the CEMB.

### 2.5 Experimental design and treatment

The animals were distributed in five groups of the individuals, each group having five males and five females randomly picked, and body weight did not differ significantly. The G1 group contained animals receiving standard diet as prescribed by the CEMB animal housing facility, G2 contained animals receiving 30% non-GM diet, G3 received 30% GM diet, G4 received 50% non-GM diet and G5 received 50% GM diet.

### 2.6 Observations (clinical), weight gain and feed intake

Animals were observed daily for mortality, morbidity, toxicity and abnormal behaviour. Daily feed consumption was estimated weekly as the amount of feed supplied minus the amount of feed left (in grams). Body weight gain was also measured at weekly intervals.

### 2.7 Pathological inspection

Before sacrificing, weight measurement was recorded for all rats. During necropsy, a complete gross visual pathological examination was done for each rat. For histopathological assays, reproductive organs (testes and ovaries) were trimmed and fixed in 10% neutral buffered formalin (NBF) in a 20:1 ratio (formalin to tissue). Fixative was removed by rinsing with 1X PBS, followed by dehydration in a series of graded ethanol, cleared with xylene, and finally paraffin embedded. Paraffined tissues were sectioned to 5 μm thickness using a microtome (Microme HM 340-E, Epredia), sections were placed on slides and stained with haematoxylin and eosin (H&E) for observation under a microscope (Leica DM2000).

### 2.8 Serum sex hormone levels

After finalizing the experimental treatments, rats were subjected to fasting for a period of 12 hours prior to blood sampling. Whole blood was collected from the rat heart without the addition of anticoagulant. Sera were obtained after centrifugation of blood at 12,000 rpm for five minutes in a microcentrifuge (Eppendorf 5415 R). Sera were assayed for measurement of testosterone in male rats, estradiol and progestin in female rats, while follicle-stimulating hormone (FSH) and luteinizing hormone were recorded for both genders, using a rat sex hormone ELISA kit (Glory Science Co., Ltd).

### 2.9 Analysis of gene expression

To evaluate the relative gene expression of fertility-related genes, total RNA was purified from testes and ovaries and converted to cDNA to analyse gene expression through qRT-PCR. After the 90 days feeding trial, ovaries and testes were removed and cut into 2.0 cm segments, placed in RNAlater (Invitrogen) and stored at 4°C overnight. RNA was isolated using the Trizol method (Invitrogen) as described earlier by Salisu et al., [37]. The extracted RNA was resuspended in RNase-Free water and then quality and integrity were evaluated using the NanoDrop 1000 spectrophotometer (Thermo Fisher Scientific). The RNA (1 μg) was converted into cDNA using the RevertAid first-strand cDNA synthesis kit (Thermo Scientific).

Four important genes with critical roles in male sexual development [SOX-9 (SRY-box 9), FGF9 (fibroblast growth factor 9), CATSPER1 (cation channel sperm associated 1), and USP9Y (ubiquitin specific peptidase 9 Y-linked)] [39–43] were selected for gene expression studies. Likewise, five important genes involved in female sexual development [i.e WNT4 (Wnt family member 4), FIGLA (Folliculogenesis-specific basic helix-loop-helix), FOXL2 (Forkhead box protein L2), GDF9 (Growth/differentiation factor 9), and NOBOX (NOBOX oogenesis homeobox)] [44–47] were selected for gene expression studies in females. The GAPDH gene was used as an internal control to normalize the data. All gene sequences were retrieved from the nucleotide database of NCBI. Evaluation of each sequence was done by NCBI Blast searches. For qPCR, primers were designed using Primer premier software (Version 6.25). The list of primer sequences used in this study is given in Table 2. Reaction mixtures consisted of 0.5 mM of each primer, 10 μl of SYBR Green qPCR Master Mix and cDNA as template. The final volume was adjusted to 20 μl with nuclease-free water. The samples were run in three technical replicates and three biological replicates. Amplification parameters included initial denaturation at 94°C for 4 minutes, followed by 40 amplification cycles of 40 s at 94°C, annealing at 60°C for 35 s and extension at 70°C for 40 s. Finally, melting curve analysis with a gradient of 60°C to 95°C was made to check the specificity of amplicon and non-appearance of primer dimers. Relative gene expression data were estimated using the 2^−ΔΔCT^ method as earlier described by Guertler et al., [48].

**Table 2:** Details of primer pair used for the gene expression study.

Primer name	Primer Sequence 5’-3’	Product length (bp)	Gene Accession number
Sox9.fwd	GCACATCAAGACGGAGCAACTG	157	NM_080403.1
Sox9.rev	GTTCTGGTGGTCGGTGTAGTCA		
Fgf9.fwd	TGCTTCATGCGGTGGGTTCTT	143	NM_012952.1
Fgf9.rev	CAGGAGGACCAGCCAGTAAGTG		
Catsper1.fwd	GGACCCACAAGAAGGCACACT	147	XM_008760198.2
Catsper1.rev	TCAGGCAGACCACGAGGAAGA		
Usp9y.fwd	TGGAATGGCTTGGAGACGAACTTG	197	JF827152.2
Usp9y.rev	GGCATCTTGGTCATCAGTGTCCTC		
Figla.fwd	TGGAACACCTGAAGCGGAGCA	183	XM_575589.6
Figla.rev	TGAGTCTACAGATGGCGGCAAGT		
Foxl2.fwd	TTGTCGCCTCTGCTGTTCCT	165	XM_003750571.4
Foxl2.rev	GCCTCGGACACTTCGATTGC		
Gdf9.fwd	CTCTGATAGGCGAAGTGAGACC	138	NM_021672.1
Gdf9.rev	CGGACGGTATTGTAGAGGTGAC		
Nobox.fwd	GCTGACTTCGGACCAGACTCT	155	NM_001192013.1
Nobox.rev	GCGGAGGTAGCACTTGAGGA		
Wnt4.fwd	CCATTGAGGAGTGCCAATACCA	142	BC098752.1
Wnt4.rev	GCCACACCTGCTGAAGAGATG		
GAPDH.fwd	CGGCAAGTTCAACGGCACAGT	128	AF106860.2
GAPDH.rev	CGACATACTCAGCACCAGCATCAC		

Note; bp; base pair, Tm: annealing temperature

### 2.10 Statistical analysis

Statistical analyses were performed using the Graph Pad Prism software (v 7.0). Data of weekly body weight gain, feed consumption, gene expression and serum sex hormones were analysed using one‐way ANOVA. The data were analysed separately for male and female groups and presented as x̅±SD. Tukey’s Multiple Comparison Test was applied to determine any differences between treatments.

## 3. Results

### 3.1 Certification and compositional analysis of diet

The aflatoxin concentration in corn seeds was below detection limits in reference control and transgenic seed sources (data not shown). Amplification by PCR revealed the presence of *cry1Ac* and *cry2Ab* transgenes (S1 Fig) whereas ELISA revealed the toxin expression in transgenic maize (S2 Fig). Proximate analysis of control and experimental diets is presented in Table 3. The table shows that formulated diets were nutritionally comparable and not significantly different.

**Table 3:** Proximate analysis of control and experimental diets (N = 3, Mean).

Ingredients (%)	Control Diet	Non-transgenic maize		Transgenic maize	
		30%	50%	30%	50%
Dry matter	90.60	91.70	92.03	91.32	91.60
Moisture	9.40	8.3	7.97	8.68	8.4
Crude protein	21.56	23.60	22.62	23.04	23.16
Crude fiber	4.80	4.00	4.25	4.75	4.25
Ether extract	9.60	8.50	9.85	8.16	9.00
Ash	6.88	7.64	7.48	7.04	7.20
NFE	46.16	46.26	44.80	45.01	44.39

Note; NFE; nitrogen free extract

### 3.2 Clinical observations, weight gain and feed consumption

**Fig 1:**
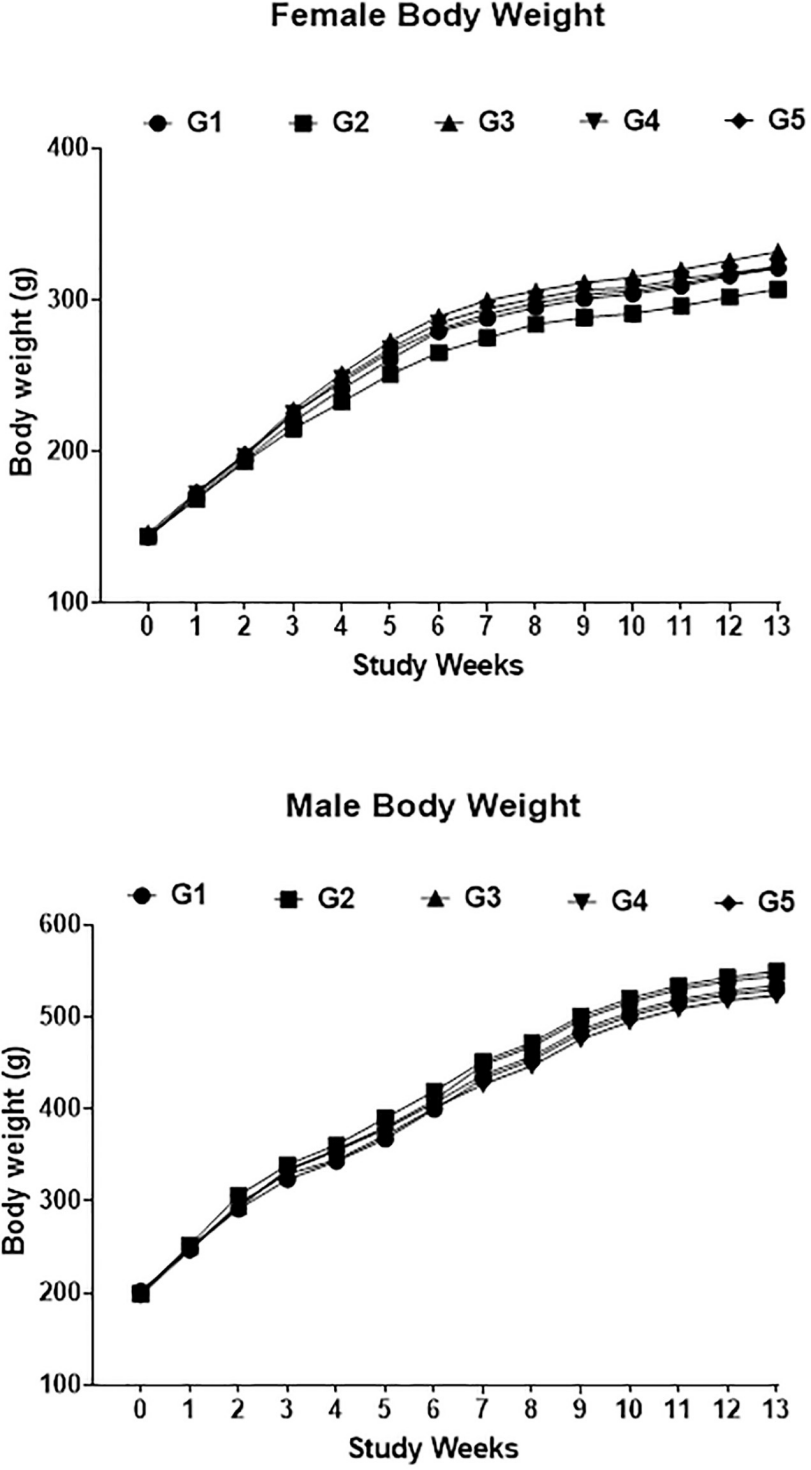
Graph showing the body weight gain comparison between the groups of male and female rats. G1: Control diet-fed rats, G2: 30% non-GM diet-fed rats, G3: 30% GM diet-fed rats, G4: 50% non-GM diet-fed rats, G5: 50% GM diet-fed rats.

**Fig 2:**
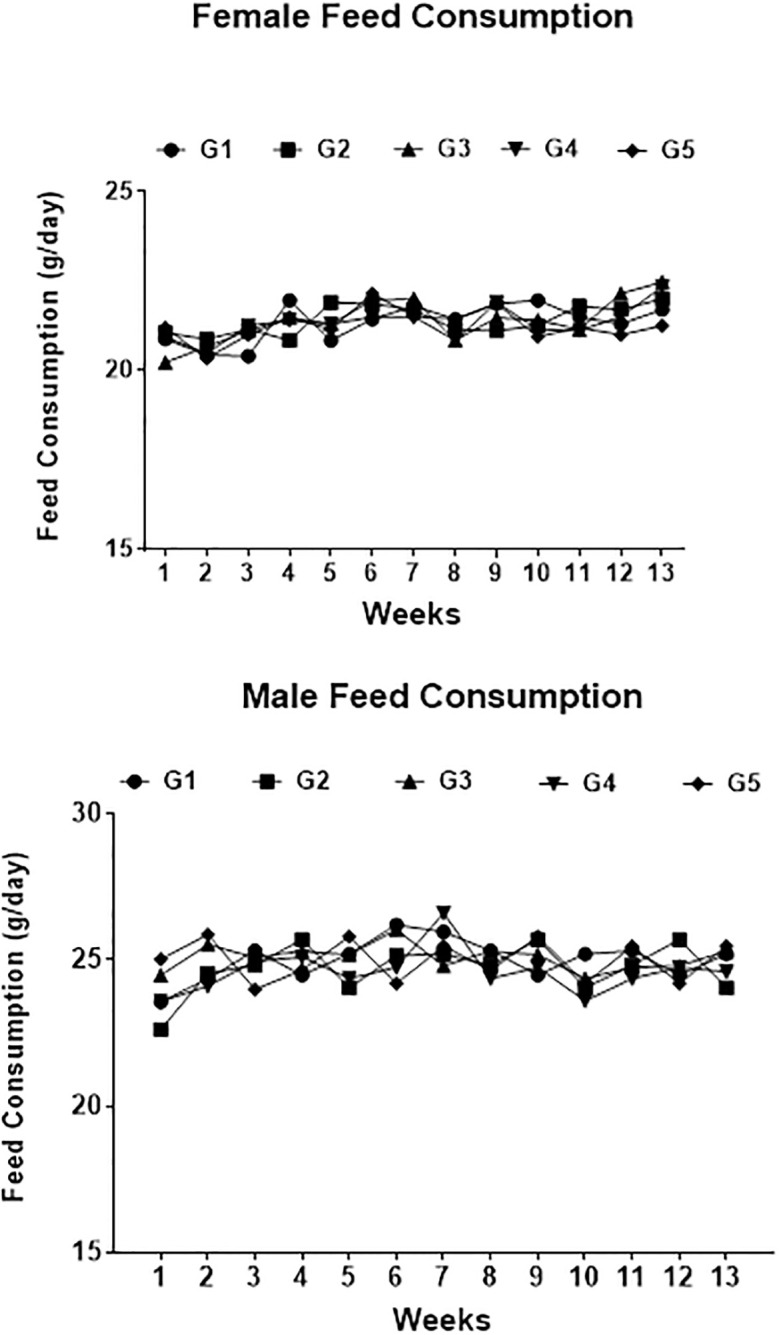
Graph showing feed consumption comparison between the groups of male and female rats. G1: Control diet-fed rats, G2: 30% non-GM diet-fed rats, G3: 30% GM diet-fed rats, G4: 50% non-GM diet-fed rats, G5: 50% GM diet-fed rats.

During the experiment, animals survived well and remained healthy. There was no mortality, morbidity, allergenicity, change in behaviour and other activities in any of the groups, regardless of gender. Overall weight of rats increased with time and weight gain of either sex was comparable. No significant body weight differences were recorded in dietary groups (Fig 1). No statically significant differences were found in feed consumption (Fig 2).

### 3.3 Morphology of the organs and histopathology

**Fig 3:**
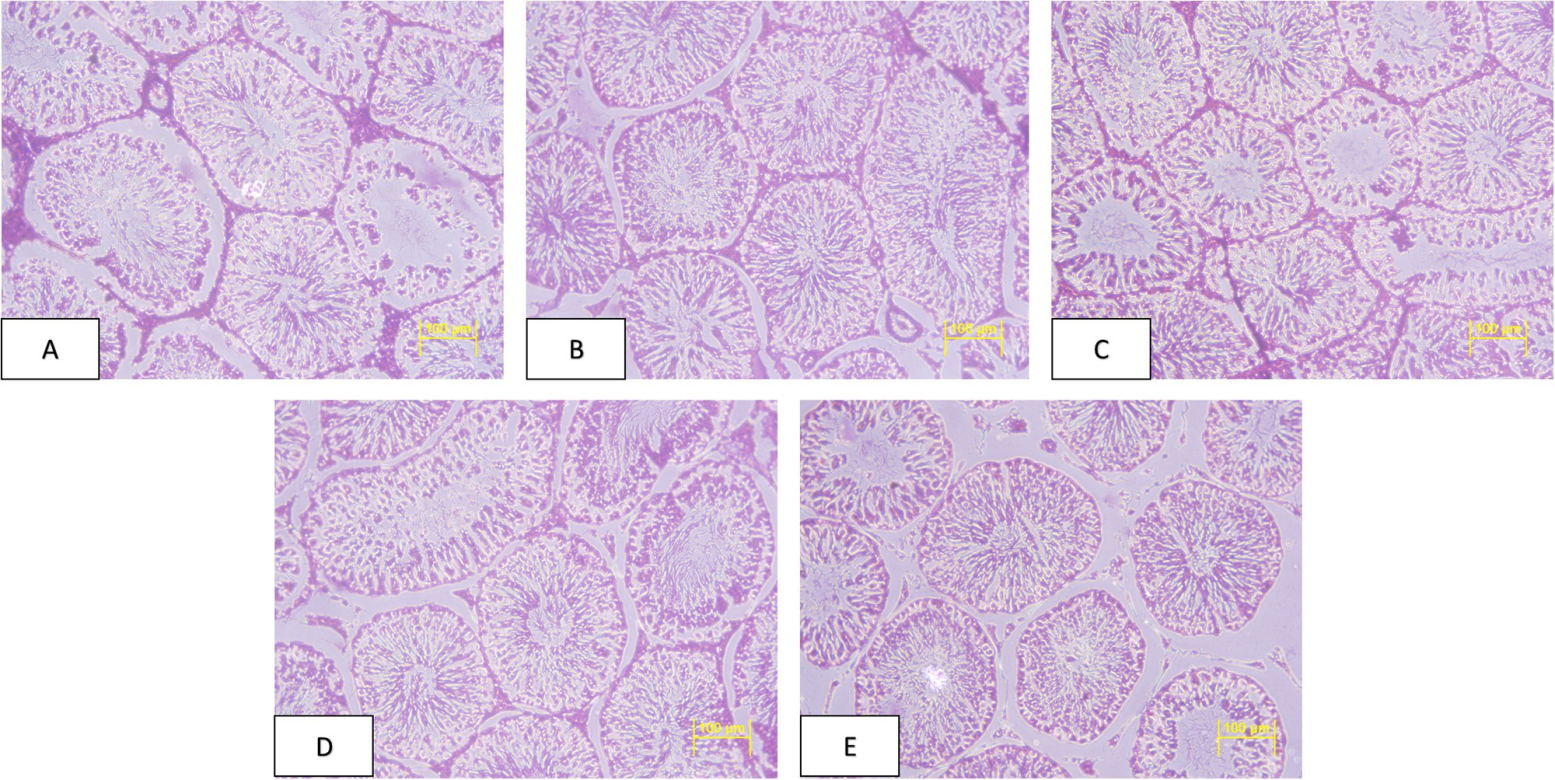
Photomicrographs of testis haematoxylin (H) and eosin (E) stained cross sections taken at x 200. A-E: Group G1-G5 male, G1: Control diet fed rats, G2: 30% non-GM diet fed rats, G3: 30% GM diet fed rats, G4: 50% non-GM diet fed rats, G5: 50% GM diet fed rats.

**Fig 4:**
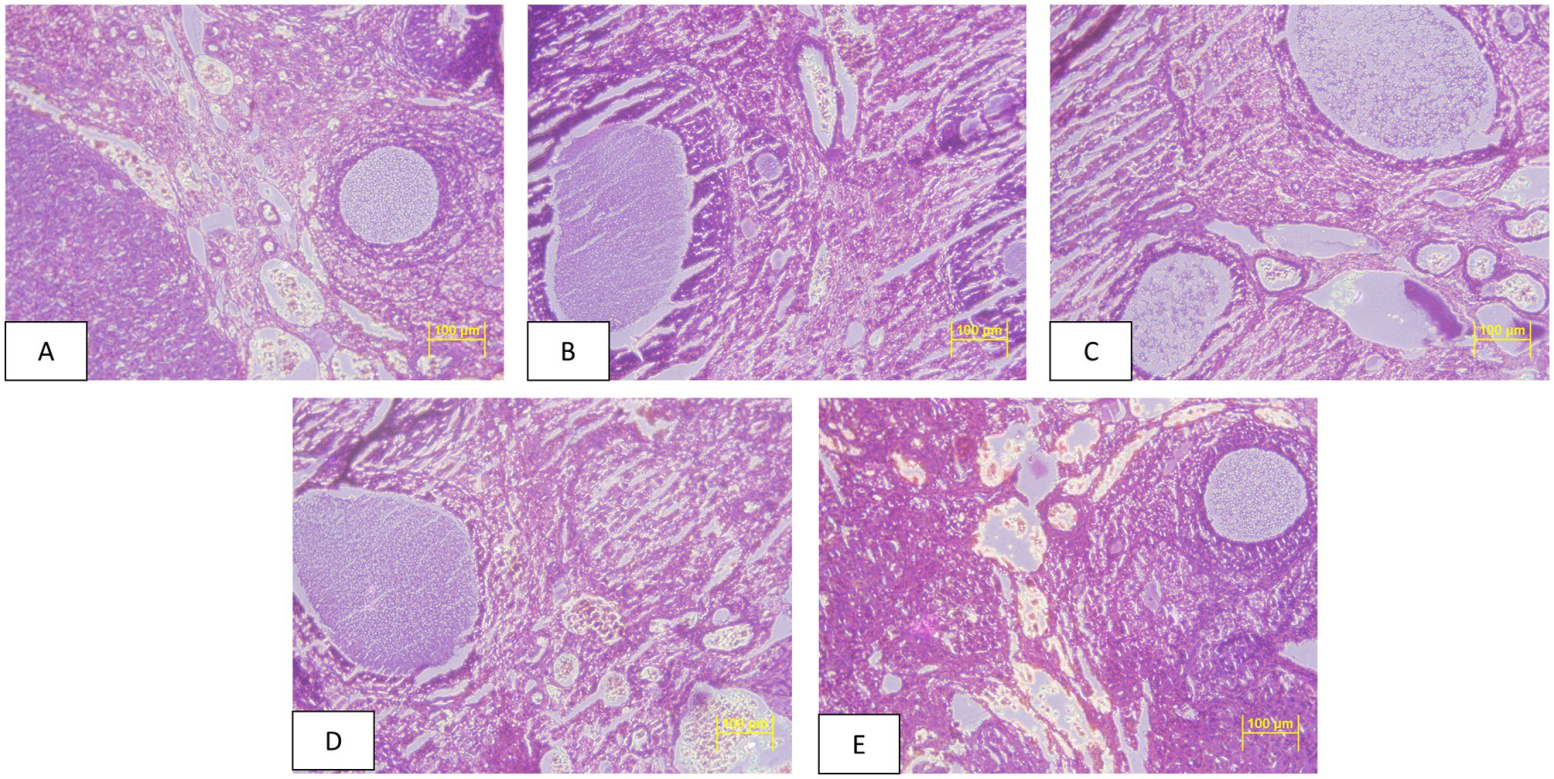
Photomicrographs of ovaries haematoxylin (H) and eosin (E) stained cross sections taken at x 200. A-E: Group G1-G5 female, G1: Control diet fed rats, G2: 30% non-GM diet fed rats, G3: 30% GM diet fed rats, G4: 50% non-GM diet fed rats, G5: 50% GM diet fed rats.

After a gross necropsy, no treatment-related changes were recorded in the morphology of the organs of animals. No treatment linked histopathology changes were recorded in tissues of the testes and the ovaries in animals under study (Figs 3 and 4).

### 3.4 Serum sex hormone

**Fig 5:**
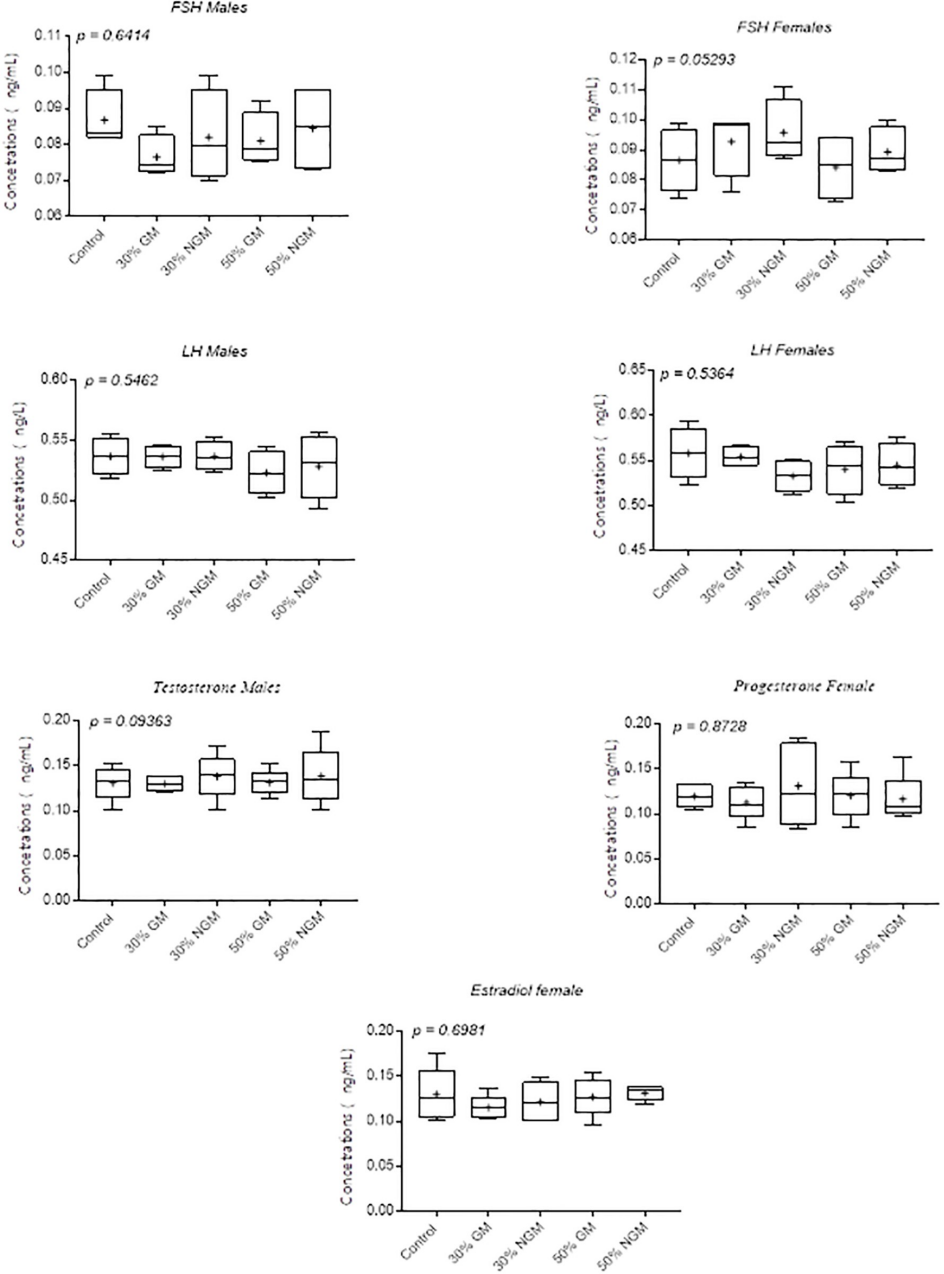
Serum sex hormones levels in various feeding groups.

Results revealed no significant (p > 0.05) variations in serum sex hormone levels of both male and female rats fed with GM feed compared to the control group (Fig 5).

### 3.5 Expression of fertility related gene in male rats

**Fig 6:**
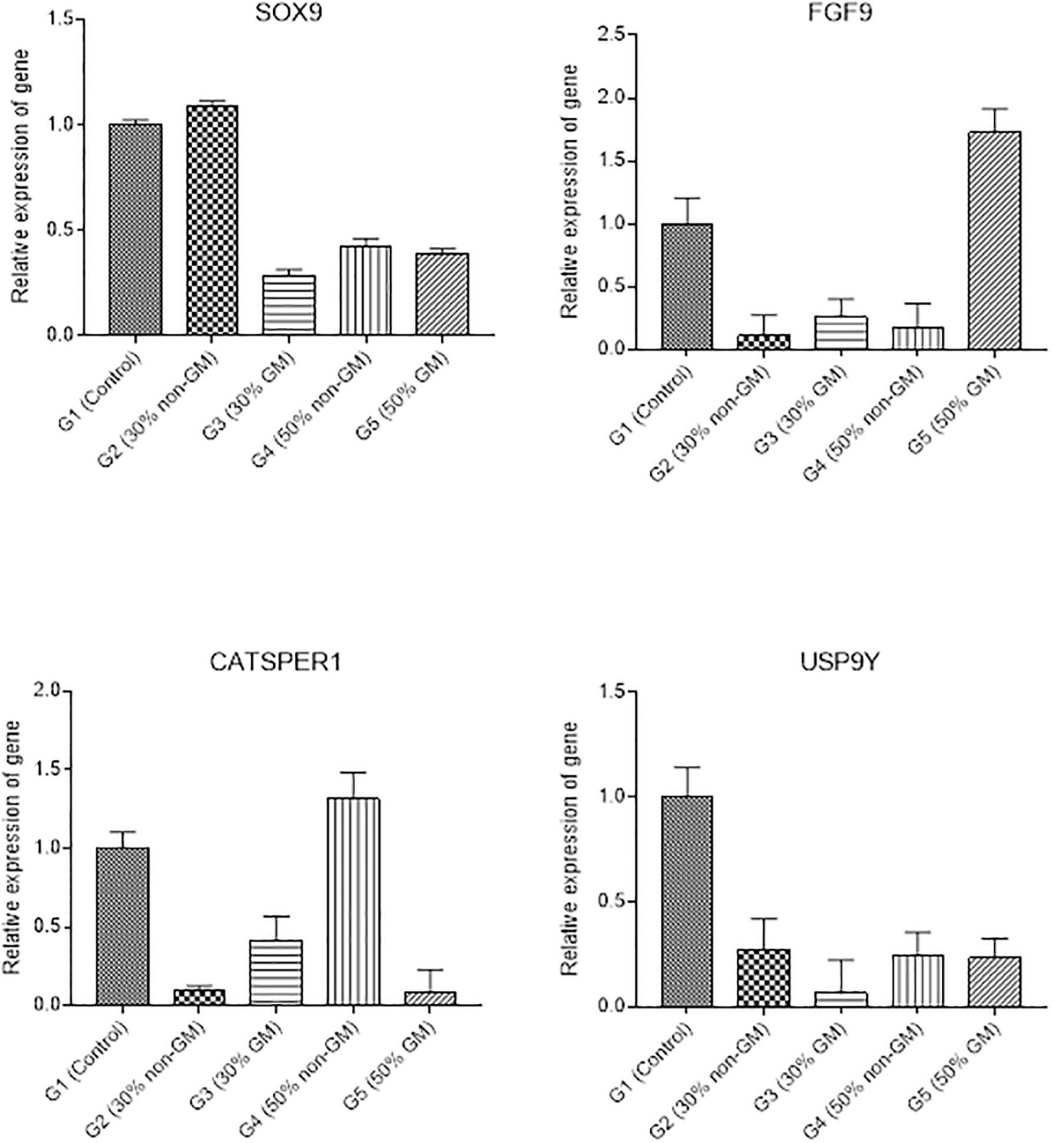
Relative expression of fertility related genes in the testis of male rats. Relative expression of fertility related genes was presented as mean ± S.D.

In testis of the male rats, relative gene expression of SOX9 was significantly different among different diet groups (Fig 6). SOX9 expression was not statistically different in G2 when compared to G1 (control) but was significantly downregulated in G3, G4 and G5. However, the SOX9 interaction among G3, G4 and G5 was statistically similar. The relative expression for FGF9 was also significantly different among experimental groups when compared to control. The FGF9 expression was significantly downregulated in G2, G3 and G4 but upregulated in G5 in comparison to control group (G1). However, the expression of FGF9 was not significantly different among G2, G3 and G4. CATSPER1 relative expression was not statistically different in G4 when compare with G1. However, its expression significantly downregulated in G2, G3 and G5 as compared to G1. The relative expression of USP9Y was significantly downregulated in G2, G3, G4 and G5 as compared to G1, but it was not significantly different between G2, G3, G4 and G5.

### 3.6 Differential expression of genes related to fertility in female rats

**Fig 7:**
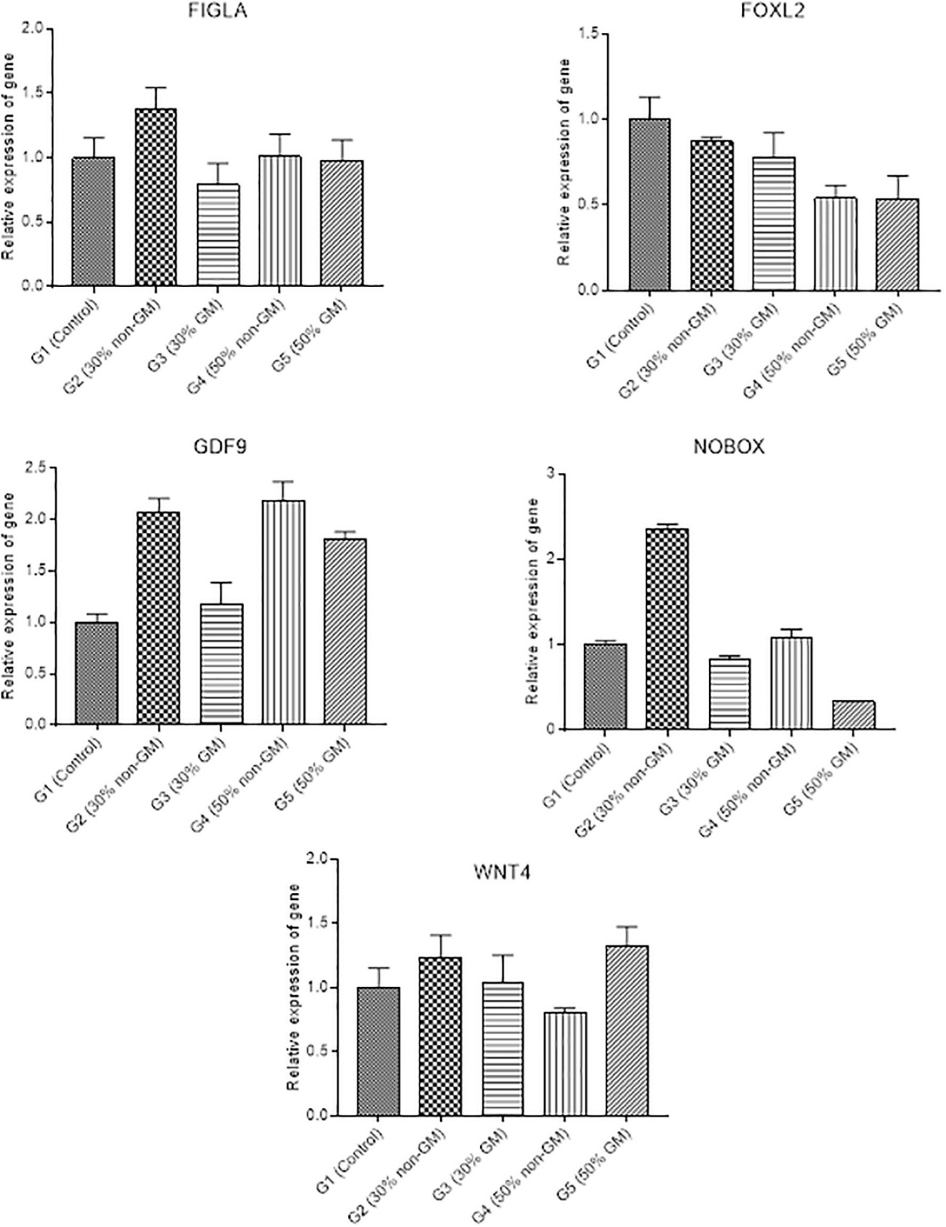
Relative expression of fertility related genes in the ovaries of female rats. Relative expression of fertility related genes was presented as mean ± S.D.

Exposure of animals to different diet groups did not result in any statistically significant variations in the expression levels of FIGLA, FOXL2 and WNT4 in the ovaries of the female rats (Fig 7). The expression of GDF9 was not statistically different in G3 compared to G1 but was significantly upregulated in G2, G4 and G5. Moreover, relative expression of NOBOX did not differ significantly in G3, G4 when compared to that of G1, but was significantly upregulated in G2 and downregulated in G5 treatment.

## 4. Discussion

Modern agriculture is aiming at the development of genetically engineered crops that can express novel and desired traits having advantages over conventional crops in terms of improved nutritional profile, combating power against non-biological stress, pests, diseases, and increased productivity [7, 49]. Transgenic Bt crops have been widely grown for almost four decades for insect control without reporting any harm to human health [50]. Despite benefits of GM and Bt crops, still there are some concerns about their safe use and safety of GM food consumption [9–11]. Rigorous risk assessment analysis of GM crops/diets on model animals coupled with performance and toxicological evaluation for substantial equivalence is recommended to address these concerns [51, 52].

However, some regulatory authorities have suggested to conduct studies that include the highest GM ingredient concentration without disturbing the overall balance of diet [EU, 53]. The scientific authorities [FAO/WHO, 14, 15] recommended 90 days feeding trials to determine effects of GM foods. Thus, a 90-day toxicology study including both low and high concentrations of GM transgenic rice was conducted on Sprague–Dawley rats to evaluate the potential physiological and biochemical effects of long-term consumption of transgenic diet [18]. Another study used GM maize at low concentration of 11–35% [22, 33]. Results from these studies did not reveal any meaningful biological difference in the health of GM-fed animals compared to controls. Similarly, some studies incorporating GM and non-GM material at high concentration in the diet of animals did not reveal any adverse effects [18, 54]. Several studies have been conducted on biosafety assessment of GM rice [55, 56], cottonseed [37], tomato [57], soybean [23, 58] and maize grain [53, 54, 59].

The present study assessed detrimental effects of GM maize (c*ry1Ac* and c*ry2A*) on Wistar rats. Several studies indicate that insertion of exogenous DNA in plants does not affect the seed nutritional value [60, 61]. In agreement with this observation, the nutritional composition of the diets in this study, including Bt maize up to 50% (w/w), was comparable and consistent with previous findings demonstrating substantial equivalence [25, 62]. These results are also in agreement with the earlier report of Salisu et al., [37] who reported no harmful effect of Bt diet (*cry1Ac*, *cry2A* and *cp4epsps*) on haematological indices of rabbits [38]. Similarly, no variations were noticed in nutrition composition of cottonseed having triple gene insect-resistant Bt cotton [63].

Allergenicity, animal body weight gain and feed consumption are precise predictors of toxicity in biosafety studies [64]. There was no sign of allergy or any toxicity found by clinical observation in this study. Similarly, no differences in clinical performance variables and histopathological responses along with no allergic reaction, mortality and morbidity were reported when rats were reared on transgenic Bt rice expressing a fused insect-resistant gene (*cry1Ab*/*cry1Ac*) for 90 days at 60% inclusion of GM rice in the animal diet [20]. In another study, exposure to lysine-rich transgenic maize at 30% (w/w) and 76% (w/w) concentration for 90 days did not induce allergic reactions [50]. In contrast, some studies have reported hepatorenal toxicity in rats feeding on herbicide-tolerant GM maize [65].

The current study revealed no significant differences in weight gain of animals feeding on GM versus non-GM diets. The results agree with an earlier study by Wang et al., [20] where Bt rice diet was fed to rats for 90 days and no difference in weight gain was recorded. Similar results were also reported when rats were fed with transgenic lysine-rich maize grain at 30% (w/w) and 76% (w/w) concentration in feed for 90 days [54]. Additionally, a long-duration study of 78 weeks revealed no significant variations in body weight of rats when exposed to transgenic rice expressing *cry1A*c and *sck* genes at > 70% (w/w) concentration [66].

No significant differences in food consumption were noticed among the groups during the study period. These consumption results are in line with what had previously been reported by Ali et al., [67] who evaluated the sub-chronic toxicity effect of triple transgenic cotton expressing binary insecticidal genes (Cry1Ac and Cry2A) as well as herbicide-tolerant genes in Sprague− Dawley rats for 90-days. Considering that weight gain is proposed to be significantly associated with improved feed utilization [68], lack of differences in feed intake observed in our study suggest that feed conversion efficiency was comparable among all the treatments.

There were no changes found in overall morphology and histopathology analysis of reproductive organs, similar to findings by Wang et al., [20] in animals feeding on GM rice. The serum sex hormonal concentration was also similar among all dietary treatments, supporting no adverse effect of GM diet. Zhou et al., [18, 19] reported no changes in serum sex hormone when Sprague-dawley rats were fed with 70% of either transgenic rice flour with high amylose level. Likewise Wang et al., [20] also reported no modification or changes in serum sex hormonal profiles when animals were fed with transgenic Bt rice created by fusion a synthetic CryAb/CryAc gene.

Fertility related parameters have been addressed in different studies of GM safety studies [42, 45, 50], yet fertility related transcript changes were not reported previously. Our study revealed significant changes in transcript levels of some fertility-related genes in testes and ovaries, yet these changes were recorded in most group irrespective of the dietary composition of GM material. There was no association between this variation and the intake of GM feed, and could be related with other factors such as gender, genetics, environment or individual physiology [69]. Similarly, Guertler et al., [48] reported no differences in transcript levels of genes critical in inflammatory responses, cell cycle and apoptosis when 42.1% of GM maize (MON 810) was supplanted in the diet of cows for two years. Fertility related parameters have been addressed in different studies; however, fertility related transcript changes have not been reported previously. This study is novel where we investigated the effect of GM diet of fertility related genes and their expression in model organism. There should be further comprehensive investigation at the transcriptome of additional fertility related genes are needed to establish safety of GM feed.

## 5. Conclusion

This study revealed no adverse effect on Wistar rats that were exposed to GM compared to control feed for a period of 90 days. Although some variations in gene expression pattern of essential fertility-related genes were observed, these variations were irrespective of GM maize content in the diet. Our findings suggest that the addition of GM maize up to 50% in rodents’ diet is safe and could not adversely affect rat’s reproductive parameters. However, gene expression of other fertility-related gene should be investigated.

## Supporting information

S1 Fig PCR analysis of reference control and transgenic maize. **A**. PCR confirmation of Cry1Ac. Lane M: 50 bp ladder (Fermentas, USA); lane 1: positive control (Recombinant plasmid DNA harbouring the cry1Ac gene); lane 2: negative control; lane 3 and 4: reference control maize; lane 5–6: transgenic maize. **B**. PCR analysis of the cry2A. Lane M: 50 bp ladder; lane1: positive control (Recombinant plasmid DNA harbouring the cry2A gene sequence); lane 2: negative control; lane 3 and 4: reference control maize; lane 5 and 6: transgenic maize.
(JPG)

S2 Fig ELISA assay of Cry1Ac and Cry2A proteins in reference control and transgenic sample. (JPG)
