# Supplementary figures and images for: A sub-chronic feeding study of dual toxin insect-resistant transgenic maize (CEMB-413) on Wistar rats
Source: PLoS One. 2023 Aug 9;18(8):e0285090. doi: 10.1371/journal.pone.0285090 (PMC10411795; doi:10.1371/journal.pone.0285090)

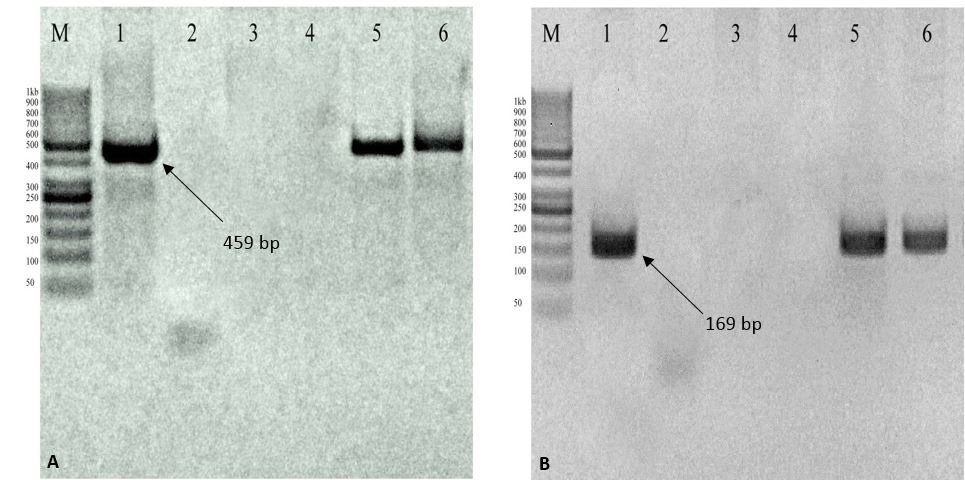

Supplement: S1 Fig — A. PCR confirmation of Cry1Ac. Lane M: 50 bp ladder (Fermentas, USA); lane 1: positive control (Recombinant plasmid DNA harbouring the cry1Ac gene); lane 2: negative control; lane 3 and 4: reference control maize; lane 5–6: transgenic maize. B. PCR analysis of the cry2A. Lane M: 50 bp ladder; lane1: positive control (Recombinant plasmid DNA harbouring the cry2A gene sequence); lane 2: negative control; lane 3 and 4: reference control maize; lane 5 and 6: transgenic maize. (JPG) [file pone.0285090.s001.jpg]

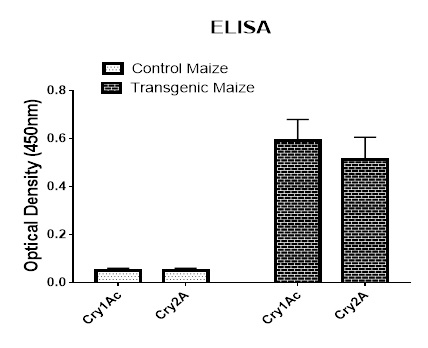

Supplement: S2 Fig — (JPG) [file pone.0285090.s002.jpg]
